# Avances y retos de la revista Anales del Sistema Sanitario de Navarra. Desempeño editorial en 2024

**DOI:** 10.23938/ASSN.1121

**Published:** 2025-04-25

**Authors:** Clara Bermúdez-Tamayo, Carmen Beorlegui

**Affiliations:** 1 Universidad de Granada Granada España; 2 Instituto de Investigación Biosanitaria ibs.GRANADA Granada España; 3 CIBER de Epidemiología y Salud Pública (CIBERESP) España; 4 Gobierno de Navarra Departamento de Salud. Servicio de Planificación, Estrategia Sanitaria e Investigación Pamplona España

En el editorial del volumen 45(2) de abril de 2022^1^, y con ocasión del 25 aniversario de la revista Anales del Sistema Sanitario de Navarra, explicamos los cambios experimentados por la revista en los últimos años y la decisión abandonar la publicación impresa cuatrimestral para optar por la publicación en formato solo electrónico, manteniendo la periodicidad. Este cambio venía de la mano de un reto: asegurar la preservación del material publicado, a fin de mantener la indexación de la revista en PubMed.

Optamos por su depósito en PubMed Central, lo que implicó actualizar las políticas de la revista para superar la requerida evaluación. Y también asumimos el reto de presentar la revista a la evaluación de calidad editorial de las revistas científicas españolas. Un año más tarde, en junio de 2023, Anales del Sistema Sanitario de Navarra obtuvo el sello que otorga la Fundación Española de Ciencia y Tecnología (FECYT), con mención en buenas prácticas editoriales en igualdad de género; actualmente nos encontramos en proceso de renovación del mismo.

En esta ocasión presentamos un balance de la actividad desarrollada por la revista durante el año 2024, con el propósito de evaluar los logros alcanzados y reflexionar sobre los desafíos que enfrentamos en 2025.

En los tres números del volumen 47, la revista ha acogido, además de los correspondientes editoriales, 24 artículos originales, dos revisiones sistemáticas, seis notas clínicas y tres cartas al editor. Este conjunto de publicaciones refleja tanto la diversidad de disciplinas y enfoques temáticos abordados en los envíos a la revista, como la amplitud geográfica de los mismos.

## Indicadores editoriales y tendencias históricas

A continuación, se presentan los principales datos sobre flujo de envíos, decisiones editoriales y tiempos de proceso de la revista Anales del Sistema Sanitario de Navarra durante el año 2024, contextualizándolos en las tendencias de años previos.

### Flujo de envíos

En 2024 se recibieron 182 envíos, manteniéndose por debajo de la serie histórica (>200/año) a pesar de suponer un incremento del 20% respecto a los 152 del año anterior.

Navarra envió 26 (14,3%), y 32% procedieron del resto de España, una cuarta parte de Madrid, Andalucía, Aragón y Comunidad Valenciana, que son las comunidades más colaboradoras durante los últimos años.

Hasta 2019, la procedencia de más del 90% de los envíos era nacional. Sin embargo, el porcentaje de envíos desde otros países se ha disparado en los últimos tres años, alcanzando un 54% en 2024. En la serie histórica, las contribuciones desde otros países se reducían a afiliaciones de autores aislados y a envíos realizados desde América del Sur/Central, siendo Perú y Colombia los países americanos que tradicionalmente realizan más envíos. Pero en 2022 se comenzaron a recibir envíos desde Asia que supusieron un 10% de los envíos desde otros países, contribución que aumentó al 40% en 2023 (tres envíos de China) y al 74% en 2024 (58 envíos de China). Estos datos han duplicado el número de envíos procedentes de otros países respecto de 2023 (54% frente a 26%) y, aunque son datos de un único año, se engloban en una tendencia ascendente en el tiempo.

El porcentaje de manuscritos redactados en inglés también muestra una tendencia creciente en Anales del Sistema Sanitario de Navarra durante los últimos cinco años y, a consecuencia de este cambio en la procedencia de los envíos, el dato de 2024 ha llegado a duplicar el de 2023 (50,5% frente a 20,4%).

La primera autoría fue femenina en el 54% de envíos recibidos.

### Decisiones editoriales

Durante 2024 se tomaron 174 decisiones editoriales. Se aceptaron 37 envíos, lo que representa una tasa de aceptación del 21,3%, dentro del rango de los diez últimos años, entre el 15 y el 27%, y ligeramente inferior al histórico de la revista (25%).

Se rechazaron 137 envíos (79%). La tasa de rechazo sin revisión por pares alcanzó el 59%, mejorando el dato de 2023 (51%), lo que sugiere un endurecimiento en los criterios de selección inicial y es consistente con el objetivo de la revista de mantener altos estándares de calidad y relevancia científica desde las primeras etapas del proceso editorial. En contraste, la tasa de rechazo tras la revisión por pares fue del 18%, lo que supone una disminución frente al 27% observado en 2023.

La primera autoría fue femenina en el 56% de los envíos aceptados y en el 52% de los rechazados. El 52,6% de personas expertas que revisaron los envíos fueron mujeres. Estos datos encajan en los estándares de paridad mantenidos por Anales del Sistema Sanitario de Navarra durante los últimos años.

### Tiempos de procesamiento

El tiempo medio en días hasta el rechazo del envío fue 19,1 días; en caso de rechazo directo la decisión se tomó en 8,12 días de media, mientras que si el envío pasó por rondas de revisión, el promedio aumentó a 68,38 días. Desde su recepción, los envíos aceptados tardaron 25 semanas en publicarse.

La Tabla 1 resume las principales métricas editoriales en los cinco últimos años (2020-2024), incluyendo número de envíos recibidos y aceptados, tasas de rechazo (sin revisión y tras pasar por rondas de revisión) y los tiempos promedio hasta la primera decisión editorial (pasara revisores o rechazar) y aceptación definitiva.

**Tabla 1:** Estadísticas editoriales sobre envíos aceptados y rechazados (número y %) en los últimos cinco años y global

	2024	2023	2022	2021	2020	Global*
*Envíos*						
recibidos	182	152	186	258	337	3.206 (198/año)
aceptados	37	31	33	41	68	791 (49/año)
rechazados	137	125	158	228	239	1.948 (130/año)
rechazados (sin revisión)	103	80	129	187	147	593 (125/año)
*Tasa anual (%)*						
de aceptación	21,3	19,9	17,3	15,3	22,2	25%
de rechazo sin revisión	59,2	51,2	67,4	69,5	47,9	
de rechazo tras revisión	18,3	26,9	15,3	15,2	29,9	
*Tiempo (días) desde recepción hasta*						
primera decisión editorial	18	25	23	34	50	50
aceptación definitiva	138	132	139	131	144	133

*: datos disponibles desde 2008.

A pesar de la reducción en los tiempos de decisión y aceptación, la revista enfrenta desafíos estructurales comunes en el ámbito editorial. Entre ellos, destaca la creciente dificultad para encontrar revisores y revisoras, derivada del aumento global en el número de publicaciones científicas y la presión por publicar, que afecta tanto a personas investigadoras como al sistema editorial en su conjunto.

Estos datos, junto con la evolución de las tasas de aceptación y rechazo, subrayan la necesidad de continuar trabajando en la eficiencia y sostenibilidad de los procesos editoriales, mientras se refuerza el compromiso con la calidad, la equidad y la transparencia.

## Autoría y procedencia de los artículos publicados en 2024

Los 38 manuscritos publicados estuvieron firmados por 168 personas. El número de autorías fluctuó entre 1 y 12, con un promedio de 4,42 por manuscrito; editoriales, cartas a la editora y casos clínicos presentaron menos autorías. En el 71% de los estudios originales/revisiones las afiliaciones mostraron colaboración entre distintas organizaciones de la misma o distinta comunidad autónoma/país. El 52% de autores fueron mujeres, firmando el 64% de las primeras autorías, el 424% de las últimas y el 50% de las de correspondencia; cinco manuscritos publicados (tres originales, una nota clínica y una carta) no estuvieron firmados por ninguna mujer.

El 84% de los 38 artículos publicados provino de España, más frecuentemente de Navarra (24%), Madrid (19%), Comunidad Valenciana (11%) y Andalucía (11%). El 24% de los artículos tuvo un carácter internacional, bien por incluir autorías de otros países, como Alemania, Australia y Polonia (8%), o bien por proceder de otros países (16%) como Chile^2^, Turquía^3^, Brasil (con un raro caso clínico de síndrome de Hamman^4^ y una carta a la editora) y China (con un estudio multicéntrico de cribado de cáncer de cérvix mediante genotipado del VPH en zonas rurales^5^ y otro estudio sobre marcadores en carcinoma papilar de tiroides^6^). El 32% de los artículos se publicó en inglés.

## Temáticas y disciplinas clínicas abordadas

La salud pública y los determinantes sociales ocuparon un lugar central en las publicaciones de este año. Un estudio realizado en España exploró los riesgos psicosociales entre policías, identificando factores laborales que impactan negativamente en su salud y satisfacción en el trabajo. Este trabajo subraya la necesidad de implementar estrategias preventivas para mitigar el estrés ocupacional y mejorar las condiciones laborales en los cuerpos de seguridad^7^. En el ámbito de salud ambiental, destaca un análisis sobre la exposición al ruido doméstico de población infantil que reveló su impacto en problemas emocionales y neurodesarrollo, particularmente en el trastorno por déficit de atención e hiperactividad (TDAH), destacando la importancia del entorno doméstico en la salud mental infantil^8^. Asimismo, una investigación sobre el conocimiento y la percepción del riesgo de enfermedades transmitidas por vectores mostró deficiencias significativas en el conocimiento de la población española, señalando la urgencia de fortalecer campañas de educación sanitaria y prevención^9^. Por otra parte, un estudio fenomenológico investigó cómo el diseño arquitectónico de los hospitales afecta la experiencia del parto. Los resultados destacaron que un entorno hospitalario acogedor y adaptado a las necesidades de las mujeres mejora significativamente su bienestar y el del recién nacido^10^. Por último, una investigación en escuelas infantiles de Chile cuantificó las pérdidas de alimentos durante la preparación de comidas, subrayando la importancia de implementar prácticas sostenibles en la alimentación infantil y en la gestión de recursos alimentarios^2^.

En el ámbito de la medicina clínica, destaca un estudio sobre biomarcadores relacionados con la lesión renal aguda inducida por contraste yodado, que identificó mecanismos moleculares vinculados al estrés oxidativo y la inflamación. Estos hallazgos abren nuevas posibilidades para mejorar el manejo de pacientes en riesgo^11^. Además, un análisis del papel de los microARN en el carcinoma papilar de tiroides estableció que niveles elevados de microARN-221 y reducidos de microARN-451 se asocian con características agresivas del tumor, lo que refuerza su potencial como marcadores pronósticos y terapéuticos^6^. En el ámbito de la fisioterapia se publicó un caso de una paciente con *gliomatosis cerebri* que mostró mejoras significativas en la funcionalidad física y en la calidad de vida, ilustrando la importancia de las intervenciones no farmacológicas en enfermedades raras^12^.

En el campo quirúrgico, se evaluó una técnica de cierre del sinus pilonidal sin suturas cutáneas que demostró una reducción significativa en las complicaciones postoperatorias y una baja tasa de recidiva durante el seguimiento a largo plazo^3^. Además, un estudio sobre patrones lesionales de trauma grave en Navarra identificó diferencias según sexo y mecanismo de lesión, proporcionando información valiosa para optimizar las estrategias preventivas y terapéuticas en estas personas^13^.

La innovación tecnológica también estuvo presente, con un estudio sobre los sesgos en la inteligencia artificial aplicada al diagnóstico clínico. Este trabajo reflexionó sobre la necesidad de diseñar herramientas tecnológicas inclusivas que consideren la diversidad en las poblaciones atendidas, evitando así inequidades^14^. Por otro lado, se validó una aplicación móvil para evaluar la capacidad cardiorrespiratoria en pacientes en rehabilitación cardiaca, mostrando buenos resultados en términos de precisión y utilidad práctica^15^.

## Retos y líneas de trabajo

El balance de 2024 nos impulsa a continuar fortaleciendo los estándares de calidad de publicación científica y la apertura hacia nuevas perspectivas científicas para optimizar la transferencia del conocimiento, fomentando el intercambio de ideas y la innovación del sistema sanitario.

Entre los desafíos, destaca la creciente competencia entre plataformas de publicación, impulsada por el aumento en el número de revistas y de trabajos científicos. Este contexto no solo incrementa la presión sobre las personas investigadoras para publicar, sino que también dificulta la labor de las revistas para encontrar revisores y revisoras cualificados, una tarea cada vez más compleja debido a la carga de trabajo que enfrenta esta comunidad.

Además, la rápida evolución de la inteligencia artificial (IA) en el ámbito editorial plantea oportunidades y retos, generando inquietudes éticas y técnicas en relación con el uso responsable de estas tecnologías en la producción científica.

En este contexto de transformación y competencia, Anales del Sistema Sanitario de Navarra reafirma su compromiso con la calidad, la ética y la transparencia en la publicación científica. Reconocemos la importancia de adaptarnos a las dinámicas cambiantes del entorno editorial sin perder de vista nuestra misión de promover el conocimiento en salud desde una perspectiva rigurosa e inclusiva.
